# The Epigenetic Legacy of Maternal Protein Restriction: Renal *Ptger1* DNA Methylation Changes in Hypertensive Rat Offspring

**DOI:** 10.3390/nu15183957

**Published:** 2023-09-13

**Authors:** Huijuan Jia, Moe Miyoshi, Xuguang Li, Kyohei Furukawa, Lila Otani, Katsuhiko Shirahige, Fumihito Miura, Takashi Ito, Hisanori Kato

**Affiliations:** 1 Health Nutrition, Graduate School of Agricultural and Life Sciences, The University of Tokyo, Tokyo 113-8657, Japan; 2 Graduate School of Bioagricultural Sciences, Nagoya University, Nagoya 464-8601, Japan; 3 Research Center for Epigenetic Disease, Institute of Molecular and Cellular Biosciences, The University of Tokyo, Tokyo 113-0032, Japan; 4 Department of Biochemistry, Graduate School of Medical Sciences, Kyushu University, Fukuoka 812-8582, Japan

**Keywords:** DNA methylation, epigenetics, *Ptger1*, hypertension, maternal nutrition, low protein diet, offspring, kidney, postnatal nutrition, nutrigenomics

## Abstract

Nutrient imbalances during gestation are a risk factor for hypertension in offspring. Although the effects of prenatal nutritional deficiency on the development of hypertension and cardiovascular diseases in adulthood have been extensively documented, its underlying mechanisms remain poorly understood. In this study, we aimed to elucidate the precise role and functional significance of epigenetic modifications in the pathogenesis of hypertension. To this end, we integrated methylome and transcriptome data to identify potential salt-sensitive hypertension genes using the kidneys of stroke-prone spontaneously hypertensive rat (SHRSP) pups exposed to a low-protein diet throughout their fetal life. Maternal protein restriction during gestation led to a positive correlation between DNA hypermethylation of the renal prostaglandin E receptor 1 (*Ptger1*) CpG island and high mRNA expression of *Ptger1* in offspring, which is consistently conserved. Furthermore, post-weaning low-protein or high-protein diets modified the *Ptger1* DNA hypermethylation caused by fetal malnutrition. Here, we show that this epigenetic variation in *Ptger1* is linked to disease susceptibility established during fetal stages and could be reprogrammed by manipulating the postnatal diet. Thus, our findings clarify the developmental origins connecting the maternal nutritional environment and potential epigenetic biomarkers for offspring hypertension. These findings shed light on hypertension prevention and prospective therapeutic strategies.

## 1. Introduction

The health status of individuals is influenced not only by their environment but also by the maternal nutritional status during gestation and lactation [1,2]. In fact, maternal nutrition plays a pivotal role in the risk of noncommunicable diseases (NCDs) in offspring, a concept known as the Developmental Origins of Health and Disease (DOHaD) [3,4]. DOHaD proposes that adverse conditions during fertilization, embryonic and fetal stages, as well as infancy, lead to epigenomic changes that predispose individuals to diseases. The interaction between postnatal life and the environment subsequently drives disease progression [5]. Diseases and other multifactorial conditions develop through these two stages, accumulating a substantial body of epidemiological research and animal experimental evidence in line with this concept [6,7,8,9]. Malnutrition during the fetal and infancy stages has been linked to several NCDs, including hypertension [10,11], cardiovascular disease [12], renal disease [13], neurocognitive disorders [14], and metabolic syndrome [15].

Epidemiological studies have demonstrated that maternal under- and overnutrition are associated with an elevated risk of hypertension in offspring [16,17,18]. Cohort studies have posited that maternal malnutrition during gestation increases the risk of hypertension in later childhood [19,20]. Additionally, specific nutrient deficiencies during pregnancy, such as vitamin D deficiency, may confer a predisposition for childhood hypertension [21,22]. Inadequate intake of micronutrients during gestation has also been linked to an elevated risk of hypertension in offspring, and maternal deficiencies in calcium [23], iron [24], and zinc [25] during gestation have been shown to result in elevated blood pressure in adult offspring. Epigenetic regulation of gene expression, particularly alterations in DNA methylation status, is considered a molecular mechanism through which the nutritional environment during fetal development contributes to disease formation [26]. Cofactors known as methyl donors, including nutrients such as vitamin B2, B6, and choline, play pivotal roles in this context [27]. In methyl donor diets, the incorporation of micronutrients, such as methionine, folic acid, vitamin B, and choline, provides methyl donors for DNA methylation and has shown effectiveness in hypertension studies [28].

Animal models are crucial for elucidating the molecular mechanisms underlying hypertension and allow for easy manipulation of the nutritional status, long-term follow-up, and specific organ dissection [29]. As such, animal models of malnutrition during gestation, including restricted calorie, low-protein (LP), and high-protein (HP)/low-carbohydrate diets, are widely employed to explore the mechanisms of induced hypertension [30]. The stroke-prone spontaneously hypertensive (SHRSP) rat model has extensively been employed in hypertension research due to its resemblance to human essential hypertension, the most common form of hypertension influenced by both genetic and environmental factors. Thus, utilizing the SHRSP model allows for an in-depth understanding of the onset and progression of hypertension [29,31]. Furthermore, knowledge of the genetic background of the SHRSP model enhances its relevance in hypertension research. Specific genes and genetic regions associated with hypertension and cardiovascular outcomes have been identified in SHRSP rats [32,33], thereby enabling genetic studies to be aimed at better understanding the genetic factors contributing to hypertension.

During early fetal development, the maternal nutritional environment is expected to induce changes in epigenetic regulation (histone modifications, non-coding RNAs, and DNA methylation) in offspring. If these modifications are maintained, they might trigger genetic changes that ultimately lead to disease onset [34,35]. In particular, DNA methylation is strongly retained in the genome and is of particular interest in DOHaD pathogenesis [36]. In animal models, the epigenetic modifications associated with DOHaD-related NCDs are reversible, and nutritional interventions can prevent NCD onset [30,37,38]. Hence, nutritional interventions during gestation have the potential to act as NCD prevention strategies to mitigate disease phenotype manifestation at different life stages, including the fetal, juvenile, adolescent, and adult stages.

In our previous study, we found that the exposure of SHRSP offspring to an LP diet during the fetal life stage caused significantly elevated blood pressure and a shortened lifespan in response to salt loading in adulthood. Furthermore, these effects were transferred to the next generation [39]. Additional studies have further shown that supplementation of folate in a protein-restricted diet could substantially reverse expression changes and DNA methylation alterations [40,41]. These findings raise the possibility that maternal malnutrition may impede the health of offspring by manipulating the epigenome in the context of the DOHaD theory. However, the underlying mechanisms of maternal–offspring crosstalk through epigenetic regulation remain unclear.

In this study, we explore the impact of maternal protein restriction during gestation on DNA methylation in offspring in an SHRSP rat model. We integrated methylome and transcriptome data to identify potential salt-sensitive hypertension genes using the kidneys of SHRSP pups exposed to an LP diet throughout their fetal life. Specifically, we aimed to elucidate how specific candidate marker genes are epigenetically modified as a result of intergenerational nutritional imbalances. We also sought to determine whether different nutritional interventions aid in mitigating these modifications at a later point in time.

## 2. Materials and Methods

### 2.1. Study Design

Nine-week-old female and male SHRSP/Izm rats were procured from the Disease Model Cooperative Research Association (Kyoto, Japan) [42]. After a week of acclimation to the prescribed environmental conditions, virgin female SHRSP rats were mated with a single male stud, and pregnancy was confirmed by the presence of a vaginal plug, as previously described [39]. Subsequent experiments were carried out according to the study design in Section 2.1.1 and Section 2.1.2. Rats were housed in a controlled environment with a temperature of 22 ± 1 °C, humidity of 60 ± 5%, and a 12 h light/dark cycle (08:00 to 20:00) [43]. This study received approval from and was conducted in strict adherence to the guidelines of the Animal Usage Committee of the Graduate School of Agricultural and Life Sciences at The University of Tokyo (Approval No. P09-376).

#### 2.1.1. Prenatal Low-Protein Diet

Pregnant dams were randomly assigned to receive either a control diet (mCN) or a low-protein diet (mLP) during pregnancy (Supplementary Table S1). After delivery, all dams were switched to a control diet (CN; Oriental Yeast, Tokyo, Japan). Next, male pups were separated from their dams at the fourth postnatal week and provided ad libitum access to drinking water and a control diet. Finally, 5-day-old (D5-CN or D5-LP), 10-day-old (D10-CN or D10-LP), and 4-week-old (D28-CN or D28-LP) male offspring were euthanized, and their kidneys were collected.

#### 2.1.2. Postnatal Low and High-Protein Diets

During pregnancy and lactation, rats were housed as described in Section 2.1.1. At the fourth postpartum week, male offspring were separated from their dams and provided ad libitum water and CN, LP diets, and high-protein diets (HP) (Supplementary Table S2) for two weeks. They were then euthanized, and their kidneys were recovered.

### 2.2. DNA Extraction and Methylome Analysis

DNA extraction was performed using the DNAiso Reagent (Takara Bio Inc., Shiga, Japan) following the manufacturer’s instructions. The concentration of the extracted DNA was determined using the NanoDrop^®^ 1000 Spectrophotometer (NanoDrop Technologies, Wilmington, DE, USA), and its quality was confirmed at OD_260/280_ > 1.8 and OD_260/230_ > 1.8. Electrophoresis (100 V, 20 min) with a 1.2% agarose gel was used to check for DNA degradation. DNA concentration was measured again using Qubit (Thermo Fisher Scientific, Waltham, MA, USA). The extracted DNA was stored at −80 °C until further analysis.

Whole-genome bisulfite sequencing was conducted using the post-bisulfite adapter tagging strategy. The sequencing library was prepared as described by Miura et al. [44], with single indexing. Sequencing was performed with a paired-end mode for 2 × 150 cycles using the HiSeq X Ten system (Illumina, San Diego, CA, USA) by Macrogen Japan Corp. (Tokyo, Japan). The basic statistical variables for the methylome data are summarized in Supplementary Table S3.

Data were analyzed using the following tools: Quality control by Fastqc (http://www.bioinformatics.babraham.ac.uk/projects/fastqc/, accessed on 2 December 2018), Mapping by Bismarck (reference BN/SsNHsdMCW rat) (http://www.bioinformatics.babraham.ac.uk/projects/bismark/, accessed on 10 December 2018), Alignment and Extraction of Methylation Site by SeqMonk (http://www.bioinformatics.bbsrc.ac.uk/projects/seqmonk/, accessed on 28 January 2019), and Reference Genome (Rnor_6.0, *Rattus norvegicus* (Norway rat). To extract the DNA methylation sites, CpG sites were narrowed down using a cut-off of *p* < 0.1 in the windowed replication test. The genes in the vicinity of the sites were then extracted.

### 2.3. Bisulfite Sequencing

For bisulfite sequencing analysis, rats with body weights close to the mean were selected from each group (*n* = 3). In short [45], renal genomic DNA was subjected to bisulfite conversion using the BisulFlashTM DNA Modification Kit (Epigentek, Farmingdale, NY, USA), and then amplified using EpiTaqTM HS (for bisulfite-treated DNA) (Takara Bio Inc.) along with prostaglandin E receptor 1 (*Ptger1*) primers (Supplementary Table S4). The amplified PCR products were separated via 1% Agarose-ME gel electrophoresis (Nacalai Tesque, Inc., Tokyo, Japan) and subsequently purified using the Wizard SV Gel and PCR Clean-up System (Promega, Madison, WI, USA). These purified products were ligated into the pGEM-T Easy Vector (Promega) and transformed into *Escherichia coli* DH5α competent cells (Sigma-Aldrich, St. Louis, MO, USA). Plasmid DNA isolation was performed using the Gene EluteTM Plasmid MiniPrep Kit (Sigma-Aldrich). Commercial sequencing was carried out by Eurofins Genomics (Tokyo, Japan), and the obtained sequencing data were analyzed using the Quantification Tool for Methylation Analysis tool (http://quma.cdb.riken.jp/, accessed on 10 August 2019; RIKEN Center for Developmental Biology, Kobe, Japan).

### 2.4. RNA Extraction and qPCR

Total RNA was extracted from kidney samples using the NucleoSpin^®^ TriPrep Kit (Takara Bio Inc.) and reverse transcribed into cDNA using PrimeScriptTM RT Master Mix (Perfect Real Time; Takara Bio Inc.). The gene segments were then amplified from the synthesized cDNA using the Thermal Cycler Dice Real Time System TP800 (Takara Bio Inc.), SYBR^®^ Premix Ex Taq™ (Tli RNaseH Plus; Takara Bio Inc), and specific gene primers (Supplementary Tables S4 and S5). The 18S ribosomal RNA (18S rRNA) and beta-actin (Actb) genes were used as internal controls. Each sample was tested in duplicate for the average Ct value. Actb or 18S rRNA was used to calculate the mean normalized expression values of the target gene transcripts.

### 2.5. DNA Microarray Analysis

For DNA microarray analysis, GeneChip^®^ Rat Genome 230 2.0 Arrays (Affymetrix, Santa Clara, CA, USA) were employed. In short, the pooled renal total RNA (250 ng) from each group was reverse transcribed into double-stranded cDNA using the GeneChip^®^ 3′ IVT Express Kit (Affymetrix). Next, aliquots of the labeled DNA were combined with the hybridization solution at 45 °C for 16 h. After washing and staining, the arrays were scanned using Affymetrix GeneChip Scanner 3000 (Affymetrix). The obtained intensity files were analyzed using the statistical analysis software R version 3.3.2. For intergroup comparison, rank products were utilized after normalizing the intensity files. Expression change was selected when the false discovery rate (FDR) values were <0.2 [46].

### 2.6. Statistical Analysis

All data are presented as mean ± standard error (SE). Statistical analyses were performed using BellCurve for Excel software version 2.15 (Social Survey Research Information Co., Ltd., Tokyo, Japan). The statistical difference between the two groups (D5-CN vs. D5-LP, D10-CN vs. D10-LP, and D28-CN vs. D28-LP) at each offspring age was determined using a two-tailed unpaired Student’s *t*-test. Multiple comparison tests with two factors, namely maternal diet and offspring age, as well as with fetal protein nutrition and postnatal protein nutrition, were determined using a two-way ANOVA and Tukey’s test. Results were considered statistically significant at *p*-values < 0.05.

## 3. Results

### 3.1. Identification of Candidate Genes for Salt-Sensitive Hypertension Using D28 Pups Exposed to a Maternal Low-Protein Diet

**Figure 1:**
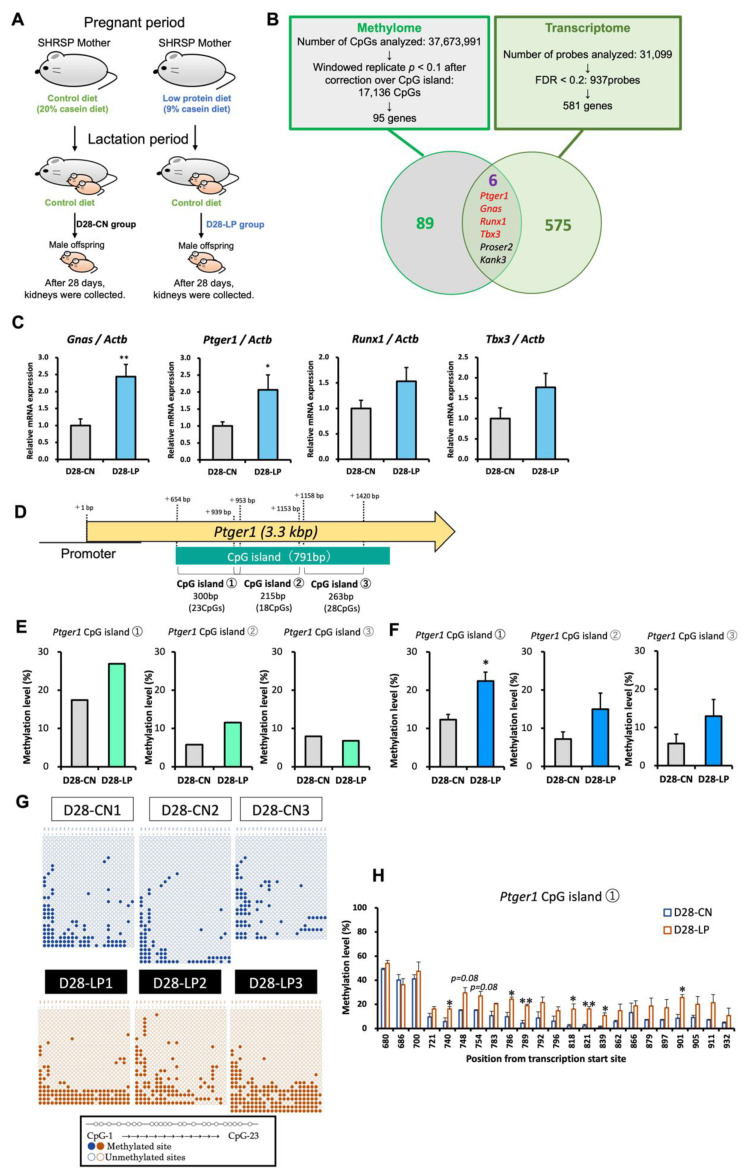
Identification of potential candidate genes and *Ptger1* CpG island DNA methylation levels in D28 pup kidneys with fetal exposure to a maternal low-protein diet. (**A**) Experimental scheme. SHRSP, stroke-prone spontaneously hypertensive pups. (**B**) Integrated methylome and transcriptome analyses were used to identify genes with coupled differential expression. Ninety-five genes were identified near the CpG sites in the methylome analysis, whereas 581 genes were identified in the transcriptome analysis. Six genes were found to show fluctuating expression in both the omics analyses. (**C**) mRNA expression of *Gnas*, *Ptger1*, *Runx1*, and *Tbx3*. The mRNA levels were normalized to those of *Actb* and expressed as fold-change values. (**D**) *Ptger1* CpG island regions and location of three primer sets (CpG①, CpG②, and CpG③). (**E**,**F**) Total DNA methylation levels of the *Ptger1* CpG island measured using (**E**) methylome and (**F**) bisulfite sequencing. (**G**) Bisulfite sequencing analysis of the *Ptger1* CpG island ①. The horizontal axis shows each evaluated CpG site. ○, unmethylated cytosine; ●, methylated cytosine. (**H**) Percentages of DNA methylation levels of CpG site in the total *Ptger1* CpG island ① were calculated using the methylome data shown in (**G**). Data in (**C**,**F**,**H**) are expressed as mean ± standard error (SE). ** *p* < 0.01, * *p* < 0.05 vs. the D28-CN group according to Student’s *t*-test. CN, control sample sizes in (**C**,**E**) were *n* = 5, and those in (**F**,**H**) were *n* = 3 rats per group.

By comparing the kidneys of SHRSP pups (D28) exposed to an LP diet (D28-LP) to those of pups exposed to a control diet (D28-CN) during fetal life (Figure 1A and Supplemental Table S6), we searched for candidate genes by integrating data from both methylome and transcriptome profiling (Figure 1B). A total of 37,673,991 CpG sites were identified from the methylome analysis, with 17,136 CpG sites retrieved at a cut-off value of *p* < 0.10, and 95 genes were identified near the CpG sites. In the transcriptome analysis, 937 probes were extracted from a total of 31,099 probes at a cut-off value of FDR < 0.2, resulting in the identification of 581 genes. Both omics analyses yielded six overlapping genes, including GNAS complex locus (*Gnas*), KN motif and ankyrin repeat domains 3 (*Kank3*), proline- and serine-rich 2 (*Proser2*), *Ptger1*, runt-related transcription factor 1 (*Runx1*), and T-box 3 (*Tbx3*) (Figure 1B and Supplemental Table S7).

Of these six genes, *Gnas* [47], *Ptger1* [48], *Runx1* [49], and *Tbx3* [50] have previously been shown to be associated with hypertension. The mRNA expression of *Gnas* and *Ptger1* in the D28-LP group was significantly increased compared with that in the D28-CN group (Figure 1C). Although *Gnas* may be involved in blood pressure regulation [51], the precise causal relationship between *Gnas,* blood pressure regulation, and renal physiology remains unclear [47,52]. Therefore, herein, we focused on the candidate gene *Ptger1*. In addition, we examined the mRNA expression of the seven genes closely associated with kidney disease that fluctuated only during the methylome analysis. However, no significant difference in the mRNA expression of these genes was found between the D28-LP and D28-CN groups (Supplemental Table S8).

### 3.2. DNA Methylation Status of Ptger1 CpG Island in D28 Pup Kidneys

The DNA methylation status of the *Ptger1* CpG island was evaluated using the bisulfite sequencing method and primers designed for three regions (*Ptger1* CpG islands ①, ②, ③), in order of proximity to the transcription start site (TSS; Figure 1D). The results showed that the total DNA methylation levels corresponded to the methylome analysis patterns (Figure 1E,F). Among the 23 CpG sites evaluated in CpG island ①, the methylation levels of seven CpG sites (TSS: +740, +786, +789, +818, +821, +839, and +901 bp) had significantly increased in the D28-LP group (Figure 1G,H). Furthermore, although the methylation levels of the *Ptger1* CpG island partial sites in CpG islands ② (215 bp, 18 CpG) and ③ (263 bp, 28 CpG) had significantly increased in the D28-LP group, total methylation levels did not differ significantly between the D28-LP and D28-CN groups (Supplemental Figure S1).

### 3.3. DNA Methylation Status of the Ptger1 Promoter Region in D28 Pup Kidneys

The DNA methylation status of the *Ptger1* promoter region, which is closely related to the regulation of gene expression, was further evaluated via bisulfite sequencing (Supplemental Figures S2 and S3). Among the evaluated CpG sites, the DNA methylation levels of nine CpG sites in the D28-LP group showed no differences from those in the D28-CN group, except for methylation of the +180 CpG site, which was significantly reduced (Supplemental Figure S3f).

### 3.4. Ptger1 mRNA Expression from D5 to D28 in the Kidneys of Pups Exposed to a Fetal Low-Protein Diet

**Figure 2:**
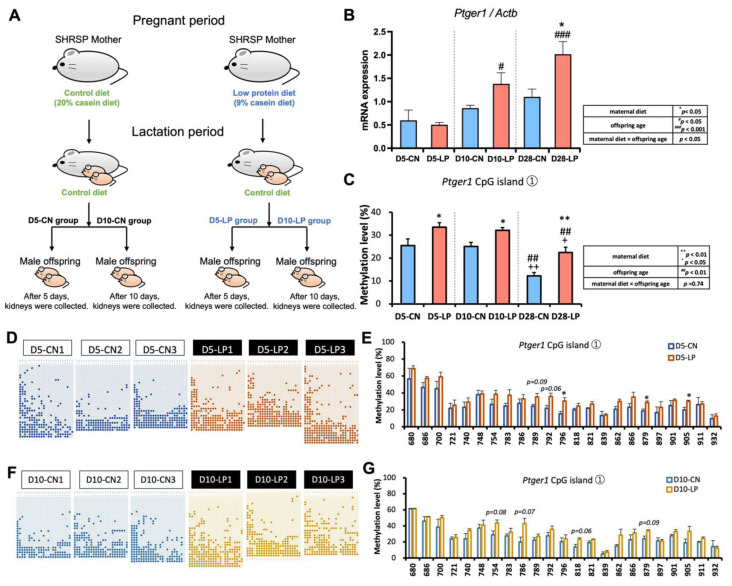
*Ptger1* mRNA expression and *Ptger1* CpG island DNA methylation levels from D5 to D28 in pup kidneys exposed to a fetal low-protein diet. (**A**) Experimental scheme. SHRSP: stroke-prone spontaneously hypertensive. (**B**) Mean normalized mRNA expression of *Ptger1* over time in pup kidneys from D5 to D28 according to maternal diet and offspring age. * *p* < 0.05 vs. corresponding CN group; # *p* < 0.05 vs. D5-LP group; ### *p* < 0.001 vs. D5-LP group. (**C**) Total DNA methylation levels of *Ptger1* CpG island ① in pup kidneys from D5 to D28 according to maternal diet and offspring age. ** *p* < 0.01, * *p* < 0.05 vs. each CN group; ## *p* < 0.01 vs. corresponding D5 group; ++ *p* < 0.01, + *p* < 0.05 vs. corresponding D10 group. (**D**,**F**) Bisulfite sequencing analysis of *Ptger1* CpG island ① in D5 and D10. The horizontal axis shows each evaluated CpG site. ○: unmethylated cytosine; ●: methylated cytosine. (**E**,**G**) Percentages of DNA methylation levels of CpG site in the total *Ptger1* CpG island ① were calculated from the bisulfite sequencing data shown in (**D**,**F**). ** *p* < 0.01, * *p* < 0.05 vs. the D5-CN group. Data are expressed as mean ± standard error (*n* = 5). Statistical analysis was performed using Student’s *t*-test (**E**,**G**) and Tukey’s test (**B**,**C**).

We tested D5 and D10 pup kidney samples to investigate any differences in *Ptger1* expression immediately after birth, as well as the changes in expression over time during the early life stages from lactation to adolescence (Figure 2A). An evaluation of the effects of maternal diet and offspring age at D5 and D10 showed significant differences in *Ptger1* expression for all factors (maternal diet, offspring age, and maternal diet × offspring age (interaction)). A significant increase was observed in renal *Ptger1* mRNA expression in the D10-LP and D28-LP rats compared with that in the D5-LP rats (Figure 2B). The restricted maternal diet also resulted in increased *Ptger1* mRNA expression, with D28-LP pups showing the most significant differences in *Ptger1* mRNA compared with that in the D28-CN pups (Figure 2B).

### 3.5. DNA Methylation Status of Ptger1 CpG Islands from D5 to D28 in the Kidneys of Pups Exposed to a Fetal Low-Protein Diet

Total DNA methylation levels of *Ptger1* CpG islands differed significantly based on either maternal diet or offspring age, but not both (interaction). The total DNA methylation level of *Ptger1* CpG islands was significantly increased in LP versus CN groups at all ages (Figure 2C). In terms of the effect that offspring age had (Figure 2C), the total DNA methylation level of *Ptger1* CpG islands was significantly decreased in the D28 groups compared to that in the D5 or D10 groups regardless of maternal diet. Furthermore, the total DNA methylation level of *Ptger1* CpG islands was significantly increased in D10-LP vs. D10-CN rats and D28-LP vs. D28-CN rats (Figure 2D).

Subsequently, the DNA methylation level of each CpG site in *Ptger1* CpG island ① was analyzed considering only the maternal diet factor at D5, D10, and D28. The DNA methylation levels of the three CpG sites (position from TSS: +796, +879, and +905 bp) in *Ptger1* CpG island ① were significantly increased in the D5-LP group compared with those in the D5-CN group (Figure 2D,E). Similarly, an increasing trend of methylation at four CpG sites (TSS: +754, +786, +818, and +879 bp) was observed in the D10-LP group compared with that in the D10-CN group (Figure 2F,G). When examining the overall DNA methylation patterns of the *Ptger1* CpG islands, the DNA methylation level at each CpG site in the D5 and D10 groups was similar to and higher than that in the D28 groups, respectively (Figure 2D–G).

No significant differences were observed in the DNA methylation status of the *Ptger1* promoter region in pup kidneys from D5 to D28 based on maternal diet or offspring age (Supplemental Figure S2).

### 3.6. Ptger1 mRNA Expression in the Kidneys of D42 Pups Exposed to Postnatal Low- and High-Protein Diets

**Figure 3:**
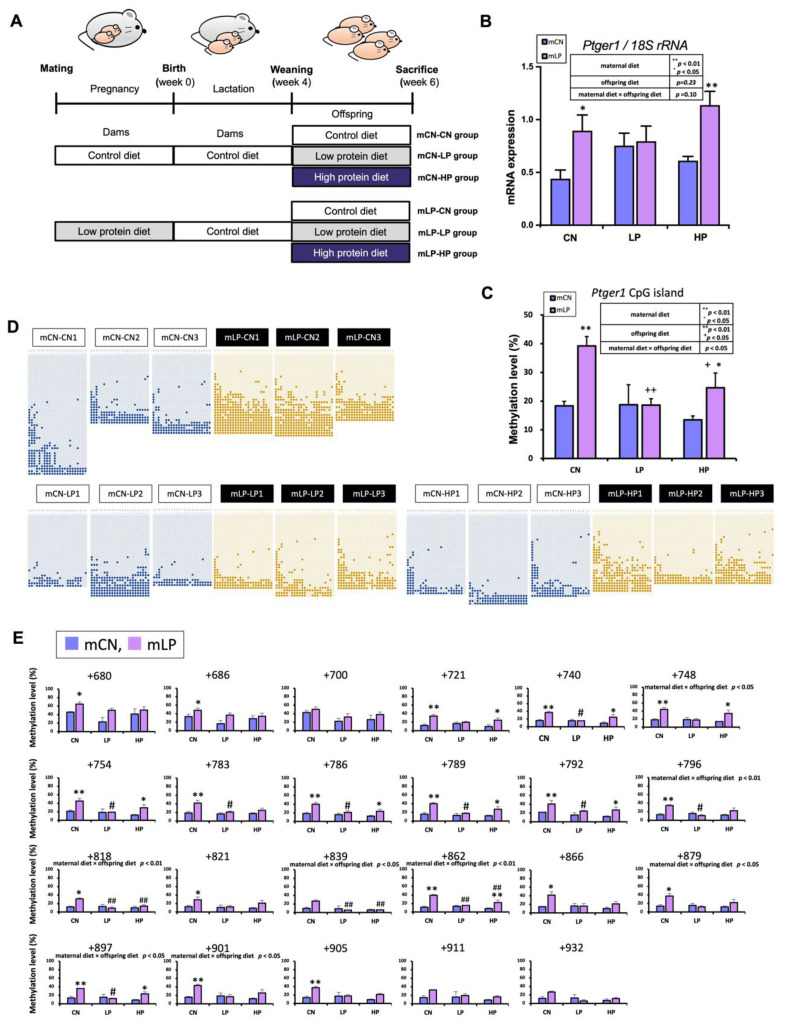
Effects of fetal exposure to a maternal low-protein diet and postnatal protein intake on *Ptger1* mRNA expression and *Ptger1* CpG island DNA methylation status in offspring kidneys (D42). (**A**) Experimental scheme. mCN/mLP: maternal control diet/maternal low protein diet; CN/LP/HP: offspring CN/LP/HP diet. (**B**) Mean normalized mRNA expression of *Ptger1* in offspring kidneys at D42 according to maternal diet and offspring age. ** *p* < 0.01, * *p* < 0.05 vs. corresponding mCN group. (**C**) Total DNA methylation levels of *Ptger1* CpG island in offspring kidneys at D42 according to maternal diet and offspring age. ** *p* < 0.01, * *p* < 0.05 vs. corresponding mCN group; ++ *p* < 0.01, + *p* < 0.05 vs. mLP-CN group. (**D**) Bisulfite sequencing analysis of the *Ptger1* CpG island at D42. The horizontal axis shows each evaluated CpG site. ○: unmethylated cytosine; ●: methylated cytosine. (**E**) Percentages of DNA methylation levels of CpG site in the total *Ptger1* CpG island were calculated from the bisulfite sequencing data shown in (**D**). ** *p* < 0.01, * *p* < 0.05 vs. each mCN group; ## *p* < 0.01, # *p* < 0.05 vs. corresponding CN group. Data in (**B**,**C**,**E**) are expressed as mean ± standard error. Statistical analysis was performed using a two-way analysis of variance followed by Tukey’s test. Sample sizes in (**B**) were *n* = 5, and those in (**C**,**E**) were *n* = 3 rats per group.

To investigate the effect of post-weaning LP or HP diets on the upregulation of *Ptger1* induced by maternal LP exposure, male offspring were assigned CN diets (mCN-CN or mLP-CN), LP diets (mCN-LP or mLP-LP), or HP diets (mCN-HP or mLP-HP) (Figure 3A and Supplemental Figure S4). Significant differences in renal *Ptger1* mRNA expression were observed after fetal exposure to an LP diet. *Ptger1* mRNA expression was significantly higher in the mLP-CN and mLP-HP groups than in the mCN-CN and mCN-HP groups, respectively (Figure 3B). There were no significant differences observed between the renal *Ptger1* levels of these groups when considering only the offspring diet factor or the combination of maternal diet × offspring diet.

### 3.7. DNA Methylation Status of Ptger1 CpG Island in the D42 Pup Kidneys Exposed to Postnatal Low- and High-Protein Diets

Significant differences were observed in the total DNA methylation levels of renal *Ptger1* CpG islands among different groups due to fetal exposure to a maternal LP diet. Total DNA methylation levels were significantly higher in the mLP-CN group than in the mCN-CN group, and in the mLP-HP group than in the mCN-HP group (Figure 3C). Correspondingly, significant differences were observed in the DNA methylation level at each CpG site (TSS: +680, +686, +721, +740, +748, +754, +783, +786, +789, +792, +796, +818, +821, +862, +866, +879, +897, +901, and +905 bp) (Figure 3D,E). The dietary protein levels of rats after weaning also affected the total DNA methylation levels of the renal *Ptger1* CpG islands, with significantly decreased levels observed in the mLP-LP and mLP-HP groups compared to those in the mLP-CN group (Figure 3C). Moreover, significant differences were observed in the methylation level at each CpG site (TSS: +740, +754, +783, +786, +789, +792, +796, +818, +839, +862, and +897 bp) (Figure 3D,E). Furthermore, fetal exposure to the maternal LP diet in the mLP-CN and mLP-HP groups significantly increased the total DNA methylation levels of the *Ptger1* CpG island compared with those in the mCN-CN and mCN-HP groups, respectively. In addition, total DNA methylation levels of the *Ptger1* CpG islands were significantly reduced in the mLP-LP and mLP-HP groups compared with those in the mLP-CN group, indicating an effect of different dietary protein levels after birth (Figure 3C). Corresponding significant differences were also observed in the DNA methylation level at each CpG site (TSS: +748, +796, +818, +839, +862, +879, +897, and +901 bp) (Figure 3D,E).

Overall, we revealed that maternal protein restriction during gestation modulates the DNA methylation status and significantly upregulates the expression of renal *Ptger1* associated with hypertension in offspring. Moreover, renal *Ptger1* DNA methylation is characterized by notable hypermethylation of CpG islands within the gene coding region and hypomethylation in promoter regions. Additionally, we observed that changes in fetal *Ptger1* DNA methylation status after birth were preserved and enhanced, strongly supporting the stable conservation of this epigenetic marker. On another note, we observed that the intake of low- or high-protein diets for 2 weeks post weaning significantly reduced DNA hypermethylation, suggesting that post-weaning low- or high-protein diets could modify *Ptger1* DNA hypermethylation arising from fetal malnutrition.

## 4. Discussion

Epigenetic modifications induced by maternal nutritional imbalances during gestation play a pivotal role in the pathogenesis of hypertension in offspring. This study was built upon the findings of our previous research in which we revealed the adverse effects of exposing SHRSP offspring to an LP diet during fetal development. This exposure led to heightened blood pressure and decreased longevity, with these impacts transmitted to the next generation. These findings raise the possibility that maternal malnutrition may influence the offspring’s health by manipulating the epigenome. However, the underlying mechanisms of maternal–offspring crosstalk through epigenetic regulation remain unclear. Our present study reveals that maternal protein restriction during gestation triggers significant DNA methylation alterations and heightened expression of renal *Ptger1*, an identified factor associated with hypertension in offspring.

Modifications in DNA methylation and the resulting gene expression changes in the kidney play a prominent role in the onset of salt-sensitive hypertension following fetal exposure to a maternal LP diet. Through a comprehensive methylome and transcriptome analysis, our study reveals the underlying molecular mechanism underpinning DNA methylation regulation in response to maternal protein restriction under different nutritional conditions. We identified *Gnas*, *Kank3*, *Proser2*, *Ptger1*, *Runx1*, and *Tbx3* as candidate genes in pup kidneys that showed fluctuating expression in both omics analyses. Fetal exposure to a maternal LP diet-induced hypomethylation and an increased expression of *Gnas*, as well as hypermethylation and an increased expression of *Ptger1*. Interestingly, the results obtained from our pups showed similar fluctuations in the renal *Ptger1* gene expression as previously reported in adult offspring (weeks 12) with salt-induced hypertension [45].

*Ptger1* encodes the prostaglandin E2 (PGE2) receptor EP1 subtype (EP1), which is involved in renal sodium retention [53,54]. PGE2 plays a pivotal role in blood pressure regulation, either by directly affecting sodium and water retention or by indirectly activating the renin–angiotensin aldosterone system [55,56]. Notably, the administration of PGE2 or EP1 antagonists can alleviate renal dysfunction in the hypertensive SHRSP model, which leads to the development of renal fibrosis with progressing hypertension [57]. Of particular relevance to our study, the *Ptger1* gene has a 791-bp CpG island containing 78 CpG sites. Within this context, our investigation delved into the DNA methylation statuses of 69 CpG sites, specifically within the region from +654 to +1420 bp relative to the TSS. Notably, our results revealed a notable hypermethylation of the *Ptger1* CpG islands in pups exposed to an LP diet during the fetal period. More precisely, we observed a statistically significant increase in methylation at 23 CpG sites located from +652 to +953 bp from the TSS (Figure 1F), with the greatest concentration between +740 and +839 bp. Interestingly, our previous study [45], focusing on adult offspring exposed to an LP diet during fetal life, also showed a significant hypermethylation pattern at 23 CpG sites between +652 and +953 bp. This consistency in methylation variability underscores the relevance of our current findings and their alignment with our previous work.

The methylation status of *Ptger1* exhibited noteworthy patterns in response to fetal exposure to an LP diet. Within the *Ptger1* CpG islands, our analysis revealed a significant increase in methylation levels. In contrast, the DNA methylation level of the *Ptger1* promoter region, spanning 10 CpG sites located between TSS −318 and +243 bp, decreased although not significantly. Moreover, a significant reduction at CpG sites concentrated around +180 bp from the TSS (Supplemental Figure S3) was observed. These findings collectively suggest that pups exposed to an LP diet during the fetal period developed a dual epigenetic profile characterized by hypermethylation within the *Ptger1* CpG islands and hypomethylation in the promoter regions. Importantly, these epigenetic changes appear to be associated with increased *Ptger1* mRNA expression. This finding is consistent with reports indicating that the DNA methylation status of promoter regions is negatively correlated with gene expression, whereas that within the genes is positively correlated with gene expression [58,59]. Taken together, these findings implicate that the methylation status of *Ptger1* may be a significant epigenetic marker in the development of salt-sensitive hypertension resulting from fetal exposure to a maternal LP diet. This insight underscores the potential relevance of *Ptger1*’s epigenetic regulation in elucidating the mechanisms underpinning hypertension in this context.

Notably, herein, prenatal LP diet exposure resulted in increased renal *Ptger1* expression between postnatal days 5 and 28 compared with that in the maternal control diet group (Figure 2B). To ensure the reliability of our findings, we assessed the expression levels of several reference genes, including *glyceraldehyde-3-phosphate dehydrogenase* (*Gapdh*), *TATA-binding protein*, *glucuronidase beta*, and *beta-actin* (*Actb)*, as evidenced by Barent et al. [60]. These reference genes, which served as endogenous controls in the kidneys of male pups exposed to a maternal LP diet, were not affected at 28, 65, and 150 days after birth. Importantly, consistent with this, neither the fetal LP diet nor age-related alterations had any impact on *Actb* expression in our study. In light of these consistent observations, our results indicate that, besides the endogenous control gene, fluctuations in *Ptger1* expression are responsible for the high renal *Ptger1* mRNA expression in male SHRSP pups exposed to an LP diet during the fetal period, and that these changes may gradually become apparent with growth. This underscores the importance of considering both temporal and dietary factors in understanding the dynamics of *Ptger1* expression in the context of maternal LP diet exposure.

During the course of our study, we observed considerable alterations in DNA methylation levels from lactation to juvenile ages (postnatal days 5 to 28) in the control and fetal LP-exposed groups, indicating that hypermethylation had occurred by postnatal day 5 (Figure 2C). Overall, the DNA methylation patterns at each CpG site located in the CpG islands of *Ptger1* were remarkably similar at postnatal days 5 and 10. However, specific CpG sites revealed substantial variations and trends from early lactation through juvenile phases, with postnatal day 28 having the largest number of changed CpG sites (Figure 1G,H). These results collectively suggest that the DNA methylation status of specific CpG sites in the *Ptger1* CpG island is not always strongly conserved in pups; however, DNA methylation of CpG sites near this region could develop with increasing age. On the other hand, within the *Ptger1* promoter region, no significant changes in DNA methylation levels were observed over time or in response to fetal exposure to a maternal LP diet, suggesting that they do not affect *Ptger1* expression during the lactation and juvenile stages. However, it is worth noting that, owing to the tendency toward hypomethylation in the promoter region of renal *Ptger1* due to fetal exposure to a maternal LP diet, alterations in the DNA methylation status of this region with growth require further investigation. Additionally, there were no differences in the expression levels of EP1 (that is encoded by *Ptger1*) in pups on postnatal days 5, 10, and 28, indicating that even if *Ptger1* mRNA expression levels fluctuate, changes in protein expression levels are not apparent in early life. In summary, these findings revealed that pups exposed to an LP diet during the fetal period were in a hypermethylated state of *Ptger1* CpG islands as early as postnatal day 5. The effects on *Ptger1* mRNA expression became apparent as early as postnatal day 10, but EP1 expression levels were unaffected up to postnatal day 28. Nevertheless, it is important to acknowledge that while our study sheds light on these associations, further experimental investigations are imperative to verify the functional role of *Ptger1* in the physiological changes observed in our study. This underscores the need for continued research in this field to advance our understanding of the underlying mechanisms.

The impact of disease predisposition stemming from the prenatal nutritional environment can interact with the postnatal environment, and the extent of this interaction can markedly influence disease risk. Specifically, the larger the gap between the prenatal and postnatal environments, the more pronounced the disease risk becomes [61,62]. For instance, a malnourished fetal environment coupled with a high-energy postnatal environment (e.g., from a high-fat diet) increases the risk of diseases, such as obesity, compared to that in pups with more nutritionally equivalent pre- and postnatal environments [63,64]. Therefore, we predicted that prenatal LP and postnatal HP diet intakes would cause variations in the DNA methylation status and gene expression due to the large nutritional gap. In contrast, pre- and postnatal LP diet intake would likely have milder effects. Our investigation validated these expectations by confirming hypermethylation of the CpG island and an increased mRNA expression of *Ptger1* in the kidney of six-week-old pups exposed to an LP diet during fetal life and subsequently fed a normal diet for two weeks post weaning (Figure 3). These results validate the conservation of renal *Ptger1* fluctuations reported here and in previous studies, as well as the possibility that the methylation of *Ptger1* is a crucial epigenetic marker of fetal LP diet-induced salt-sensitive hypertension. Notably, we observed a significant reduction in DNA hypermethylation when pups were fed either an LP diet or an HP diet two weeks after weaning. In particular, this observation contradicts our initial predictions that DNA methylation levels would remain relatively stable in pups fed an LP diet after birth. Thus, it appears that both LP and HP postnatal diets could potentially modulate or reset *Ptger1* DNA hypermethylation caused by fetal exposure to a maternal LP diet. However, the increase in *Ptger1* mRNA expression was not significantly changed by either LP or HP postnatal diets. It is worth considering that the time durations of the postnatal diets in this study may have been insufficient to detect significant changes in *Ptger1* overexpression, which may become more apparent with extended durations.

In summary, while previous studies have focused on ameliorating maternal diet-induced disease risk during gestation and lactation, this study underscores that the negative effects can also be modulated during the post-weaning stage. This finding provides additional flexibility for prevention strategies against salt-sensitive hypertension, emphasizing the importance of considering both prenatal and postnatal dietary factors in disease risk mitigation. However, it is essential to acknowledge that the short duration of our postnatal dietary interventions might have limited our ability to detect changes in *Ptger1* expression fully, highlighting the need for further research with extended post-weaning periods.

## 5. Conclusions

**Figure 4:**
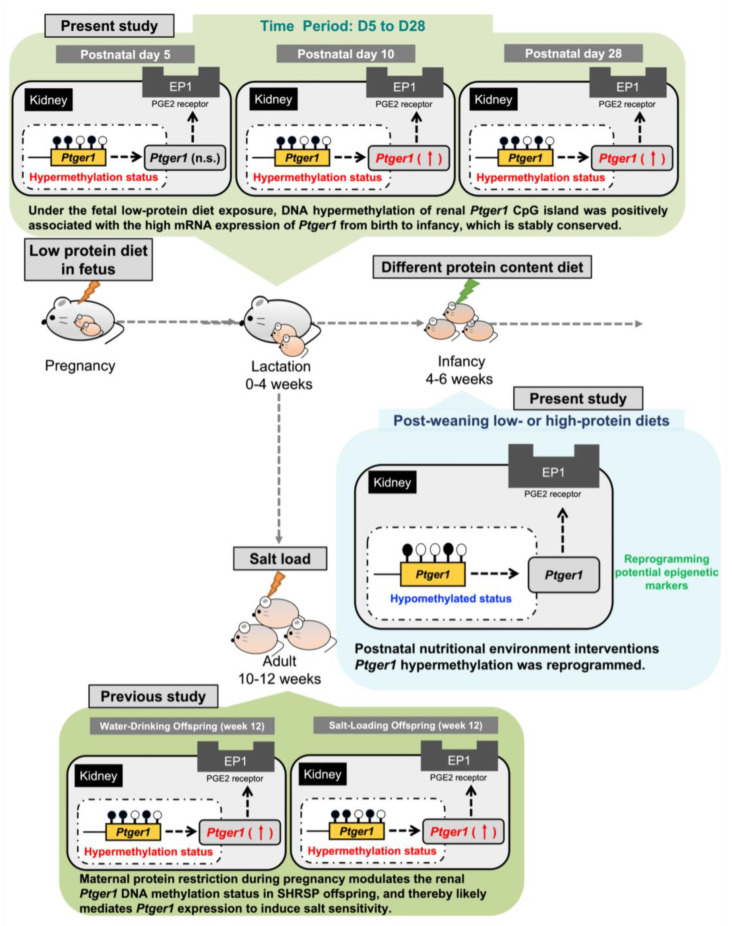
Schematic of the proposed mechanism of *Ptger1* DNA methylation and mRNA expression after fetal exposure to a low-protein maternal diet. Under fetal low-protein diet exposure, renal *Ptger1* DNA is hypermethylated and stably conserved. This epigenetic variation in *Ptger1* is associated with disease risk imprinted during fetal life and can be reprogrammed by the postnatal dietary environment.

We found that under fetal exposure to a maternal LP diet, DNA hypermethylation of the renal *Ptger1* CpG island is positively associated with a high mRNA expression of *Ptger1* from birth to infancy, which is stably conserved (Figure 4). Importantly, this epigenetic variation in *Ptger1* is linked to disease risk imprinted during fetal life and could be reprogrammed by manipulating the postnatal diet. Although further human application studies are needed, this study identified potential epigenetic biomarkers and therapeutic targets for fetal LP diet-induced salt-sensitive hypertension, as well as preventive nutritional intervention strategies that can be implemented outside of the fetal and infancy stages. Given the goal of reducing cardiovascular disease in humans, it is critical to ascertain whether similar mechanisms control the development of human offspring. This necessitates a pressing requirement to enhance our comprehension of the precise manner and contextual conditions under which interventions should be directed toward modulating the nutritional status of offspring. Therefore, further exploration of the nutritional environment and the components that can reset the disease predispositions imprinted in the fetus is expected to provide individualized nutritional guidance and lead to the development of effective diets to reduce hypertension risk.

## Data Availability

The sequence data obtained in the current study were deposited to NCBI-GEO with accession numbers GSE215329 and GSE216424.

## Supplementary Materials

The following supporting information can be downloaded at: https://www.mdpi.com/article/10.3390/nu15183957/s1. Table S1: composition of prenatal diet; Table S2: composition of low and high-protein diets in offspring; Table S3: statistical summary of methylome sequencing and the average methylation level of CN/LP group; Table S4: primer sequences for each gene; Table S5: primer sequences for the genes (*Slc14a2*, *Il1b*, *Corin*, *Hmox1*, *Gucy1b3*, and *Gdnf*) extracted in transcriptome analysis; Table S6: pregnancy-related changes in maternal body weight and feed intake, and the body weight and kidney weight of pups; Table S7: statistical data for six genes that fluctuate in both methylome and transcriptome analyses; Table S8: the genes that fluctuate in the kidneys of SHRSP pups (D28) exposed to a fetal low-protein diet; Table S9: relative ratios (fold-change) of kidney disease-related genes extracted in transcriptome analysis from D5 to D28 over time in pup kidneys. Figure S1: *Ptger1* CpG island (CpG② and CpG③) DNA methylation levels in pup (D28) kidneys of fetal exposure to maternal low protein diet; Figure S2: *Ptger1* promoter DNA methylation levels from D5 to D28 in pup kidneys exposed to a fetal low-protein diet; Figure S3: *Ptger1* promoter DNA methylation levels from D5 to D28 in pup kidneys exposed to a fetal low-protein diet; Figure S4: body weight, feed intake, and tissue weight either on a low-protein diet or a high-protein diet for 2 weeks after weaning (at week 4).
