# Supplementary material for: The Epigenetic Legacy of Maternal Protein Restriction: Renal Ptger1 DNA Methylation Changes in Hypertensive Rat Offspring
Source: Nutrients. 2023 Sep 13;15(18):3957. doi: 10.3390/nu15183957 (PMC10535296; doi:10.3390/nu15183957)
Supplement: Supplementary file 1 [file nutrients-15-03957-s001.zip › nutrients-2589508-supplementary.pdf]

Supplemental Table S1

Composition of prenatal diet. * AIN-76 prescription (Oriental Yeast Co., Ltd., Tokyo, Japan). mCN: maternal control diet; mLP: maternal low-protein diet.

Component (% w/w)	mCN (20% Casein)	mLP (9% Casein)
Casein	20.0	9.0
Corn starch	66.8	77.8
DL-Methionine	0.2	0.2
Soybean oil	5.0	5.0
Vitamin mixture*	1.0	1.0
Mineral mixture*	4.0	4.0
Cellulose powder	3.0	3.0

Supplemental Table S2

Composition of low and high protein diets in offspring. * AIN-76 prescription (Oriental Yeast Co., Ltd., Tokyo, Japan). CN: control diet; LP: low-protein diet; HP: high-protein diet.

Component (% w/w)	CN (20% Casein)	LP (9% Casein)	HP (40% Casein)
Casein	20.0	9.0	40.0
Corn starch	66.8	77.8	46.8
DL-Methionine	0.2	0.2	0.2
Soybean oil	5.0	5.0	5.0
Vitamin mixture*	1.0	1.0	1.0
Mineral mixture*	4.0	4.0	4.0
Cellulose powder	3.0	3.0	3.0

Supplemental Table S3

Statistical summary of methylome sequencing and the average methylation level of CN/LP group.

	CN	LP
**Read Number**	**754.6M**	**525.5M**
**Rate of Uniquely Mapped Reads**	**80.1%**	**80.7%**
**Rate of Reads Mapped on Multiple Sites**	**6.5%**	**6.6%**
**Rate of Reads Unmapped**	**13.4%**	**12.8%**

Average Read Depth	CN	LP
**All Nucleotides**	**24.9x**	**22.9x**
**All Cytosines**	**27.0x**	**25.0x**
**CpG sites**	**29.6x**	**26.6x**

Average Methylation Level	CN	LP
**All Cytosines**	**4.6%**	**4.5%**
**CpG sites**	**70.0%**	**70.0%**
**CHG sites**	**1.3%**	**1.2%**
**CHH sites**	**1.3%**	**1.2%**

Supplemental Table S4

Primer sequences for each gene. a-b Primer sequences used for real-time PCR of (**a**) candidate genes narrowed by integrating data from both methylome and transcriptome profiling and (**b)** The seven genes closely associated with kidney disease that fluctuated only in the methylome analysis. (**c)** Primer sequences used for the bisulfite sequencing. The number of CpGs evaluated as well as the regions that were assessed when the transcription start site (TSS) was set to +1.

a

Target gene	Primer Sequence (5' to 3')
Gnas<br>(GNAS complex locus)	Forward : AGCAGGGCAAACGAGAAGA<br>Reverse : ATGCACACCGAGAGAGATGG
Ptger1<br>(prostaglandin E receptor 1 subtype EP1)	Forward : AGTAGCTGGAGTGGGCAGCA<br>Reverse : GCTCATATCAGTGGCCAAGAGG
Runx1<br>(runt-related transcription factor 1)	Forward : GAGGCCAACAGGAGGGAAAC<br>Reverse : CACCCGGAACCGAAAACA
Tbx3<br>(T-box 3)	Forward : GGAGGAAGTGGGGTGAAAGG<br>Reverse : GTCCAACAGGCACACGAAAG
Actb<br>(beta-actin)	Rat Housekeeping Gene Primer Set, Rat Actb primer

b

Target gene	Primer Sequence (5' to 3')
Aggf1<br>(angiogenic factor with G patch and FHA domains 1)	Forward : GACTCGCAGAGTGAGTGCAG<br>Reverse : GCACAGTGTGTTCCATGTCC
Cyp24a1<br>(cytochrome P450 family 24 subfamily a member 1)	Forward : CAAACCCTGCATCGACAACC<br>Reverse : CCGAATGGGAGATGAGCGAA
Fat4<br>(FAT atypical cadherin 4)	Forward : TGTACAGCCAAACCGAGTCA<br>Reverse : TGACCAGAAGTCCACACAGG
Lhx1<br>(LIM homeobox 1)	Forward : ACTGGAGACGTTGAAGGCAG<br>Reverse : AAAGACCTCCGCAGACATGG
Pdgfa<br>(platelet-derived growth factor alpha polypeptide)	Forward : ACAAACCTGAGAGCCCATGG<br>Reverse : CGCACATTGGCAATGAAGCA
Ryr1<br>(ryanodine receptor 1)	Forward : TGAATACAACGCCTGCTCTG<br>Reverse : GCATAGGCAGTGTGATCTCG
Sema5b<br>(semaphorin 5B)	Forward : AGGTGCTCGGCCTACCTTAG<br>Reverse : CAAGAACCCGAGTTGTCTCC

c

Name	Position	Number of CpGs analyzed	Primer Sequence (5' to 3')
Ptger1 CpG island ①	＋654～＋953<br>(300 bp)	23 CpGs	Sense primer: AATATATTTGTGGTGTTGTTAATAGG<br>Antisense primer: ACCAAAAAAAACCATACAACC
Ptger1 CpG island ②	＋939～＋1153<br>(215 bp)	18 CpGs	Sense primer: ATGGTTTTTTTTGGTTTGTGTTTAT<br>Antisense primer: CACCAAATACCAAAATACTATAACTC
Ptger1 CpG island ③	＋1158～＋1420<br>(263 bp)	28 CpGs	Sense primer: ATTAGTTTTGGGTTTTTTGGAGG<br>Antisense primer: AAACAACTATAATTAAAATAATAAATAAC
Ptger1 promoter	-318～＋243<br>(561 bp)	10 CpGs	Sense primer: GTTTTATGGTTTTTAGTTTTAAGTTGTTTA<br>Antisense primer: CTTTTCAAAAATCTCCTTAACTTACC

Supplemental Table S5

Primer sequences for the genes (*Slc14a2*, *Il1b*, *Corin*, *Hmox1*, *Gucy1b3*, and *Gdnf*) extracted in transcriptome analysis.

Target gene	Primer Sequence (5' to 3')
**Slc14a2**<br>(solute carrier family 14 (urea transporter), member 2)	Forward : TGGAATGGCTCTCAGAGGAG<br>Reverse : ACGTTTGCTTACACTATGATTGGA
**Il1b**<br>(interleukin 1 beta)	Forward : TCAAGCAGAGCACAGACCTG<br>Reverse : AGAACTCAGTGCTGGCTGTG
**Corin**<br>(corin, serine peptidase)	Forward : CCTAATTTAATTAGCCAGACATCAACG<br>Reverse : CAAAACCAACCGACGGTCTTAA
**Hmox1**<br>(heme oxygenase 1)	Forward : CAGGGGAGAATCTTGCCTGG<br>Reverse : TACCACCCACCCCTCAAAAG
**Gucy1b3**<br>(guanylate cyclase 1, soluble, beta 3)	Forward : GTGGGCTTCAACGCTTTCTG<br>Reverse : CCACAAAGGCTGCCATTCAG
**Gdnf**<br>(glial cell derived neurotrophic factor)	Forward : ATGTAGTCCCAGACCCTCCG<br>Reverse : CTTACTGTGGCAGACGCTCA

Supplemental Table S6

Pregnancy-related changes in maternal body weight and feed intake, and the body weight and kidney weight of pups. a Changes in maternal body weight, weight gain, and feed intake during pregnancy. **b** Body weight and kidney weight at D5, D10, and D28 of postnatal rat. Data are expressed as means ± standard error. Sample sizes for (**a**) were n = 6-7, and for (**b**) were n = 5 rats per group. *p < 0.05 vs. the D10-CN group according to a Student's t-test.

a

Type of record	Groups	
Gestational period	CN	LP
1st day pregnancy weight (g)	175.13 ± 2.62	168.42 ± 3.60
21st day pregnancy weight (g)	261.50 ± 4.87	251.40 ± 7.08
Weight gain during pregnancy (g)	86.38 ± 3.80	82.98 ± 4.06
Feed intake (g/day)	14.73 ± 0.21	14.96 ± 0.26

b

Type of record	Groups	
5 day old rat pups	D5-CN	D5-LP
Final weight (g)	7.48 ± 0.10	7.30 ± 0.15
Kidney weight (mg)	83.32 ± 3.32	88.00 ± 5.82
10 day old rat pups	D10-CN	D10-LP
Final weight (g)	13.59 ± 0.46	14.15 ± 0.27
Final weight (mg)	159.28 ± 3.50	173.62 ± 2.54 *
28 day old rat pups	D28-CN	D28-LP
Final weight (g)	55.51 ± 0.23	57.33 ± 0.72
Final weight (mg)	569.66 ± 29.99	611.98 ± 18.85

Supplemental Table S7

Statistical data for six genes that fluctuate in both methylome and transcriptome analyses. **a** The location and number of fluctuating CpG sites extracted in methylome analysis. **b** Fold changes in gene expression obtained from transcriptome analysis.

a

Chr	Gene	Gene name	Position of CpG analyzed	Number of CpGs analyzed	Number of hypermethylated CpGs in D28-LP	Number of hypomethylated CpGs in D28-LP
Chr. 3	Gnas	GNAS complex locus	intragenic	148	36	112
Chr. 7	Kank3	KN motif and ankyrin repeat domains 3	intragenic	147	46	101
Chr. 11	Runx1	runt-related transcription factor 1	intragenic	98	45	53
Chr. 12	Tbx3	T-box 3	intragenic	88	31	57
Chr. 17	Proser2	proline and serine rich 2	intragenic	118	38	80
Chr. 19	Ptger1	prostaglandin E receptor 1 (subtype EP1)	intragenic	156	114	42

b

Gene	Gene name	ID	Fold change (LP/CN)
Gnas	GNAS complex locus	1387906_a_at	1.24
		1390263_at	1.29
Kank3	KN motif and ankyrin repeat domains 3	1373483_at	1.25
Runx1	runt-related transcription factor 1	1368914_at	1.24
Tbx3	T-box 3	1390627_a_at	1.25
		1393160_at	1.68
Proser2	proline and serine rich 2	1379057_at	1.38
		1385668_at	1.33
		1379057_at	1.38
		1385668_at	1.33
Ptger1	prostaglandin E receptor 1 (subtype EP1)	1369254_a_at	1.49

Supplemental Table S8

The genes that fluctuate in the kidneys of SHRSP pups (D28) exposed to a fetal low-protein diet. The expression of seven genes (*Aggf1, Cyp24a1, Fat4, Lhx1, Pdgfa, Ryr1,* and *Sema5b*) closely associated with kidney disease, which fluctuated only in methylome analysis, were analyzed by transcriptome analysis and real-time PCR. Data are expressed as means ± standard error (n = 5). N/A, Not Applicable. CN and LP, offspring CN/LP diet.

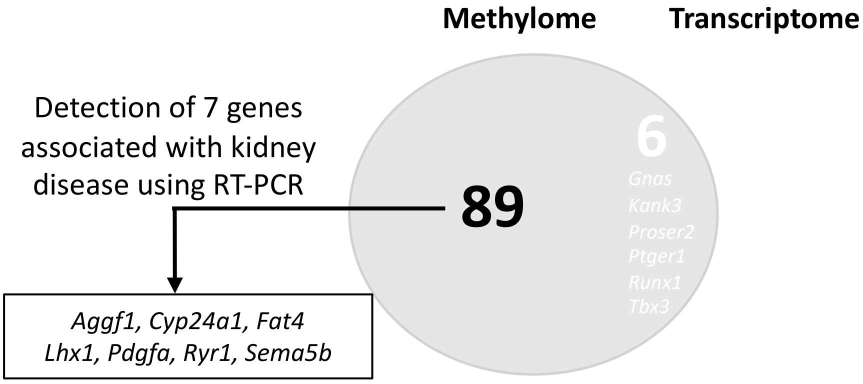

Gene	Gene name	Transcriptome		qPCR	
		ID	Fold change (D28-LP/D28-CN)	Relative mRNA expression (D28-CN)	Relative mRNA expression (D28-LP)
**Aggf1**	angiogenic factor with G patch and FHA domains 1	1372768_at<br>1396100_at	1.08<br>1.11	1.00 ± 0.16	0.79 ± 0.20
**Cyp24a1**	cytochrome P450 family 24 subfamily a member 1	N/A	N/A	1.00 ± 0.32	0.93 ± 0.48
**Fat4**	FAT atypical cadherin 4	1374643_at	1.09	1.00 ± 0.10	1.26 ± 0.34
**Lhx1**	LIM homeobox 1	1377206_at	1.06	1.00 ± 0.33	0.78 ± 0.14
**Pdgfa**	platelet-derived growth factor alpha polypeptide	1370427_at<br>1379375_at	1.04<br>0.93	1.00 ± 0.22	0.74 ± 0.08
**Ryr1**	ryanodine receptor 1	1390355_at<br>1390904_at	1.00<br>0.99	1.00 ± 0.13	0.82 ± 0.21
**Sema5b**	semaphorin 5B	N/A	N/A	1.00 ± 0.11	0.99 ± 0.20

Supplemental Table S9

Relative ratios (Fold-change) of kidney disease-related genes extracted in transcriptome analysis from D5 to D28 over time in pup kidneys.

Gene	Gene name	ID	Fold-change(D5)	Fold-change(D10)	Fold-change(D28)
**Increase (Continuation)**					
Slc14a2	solute carrier family 14 (urea transporter), member 2	1387550_a_at	1.24	1.45	1.33
**Decrease (Continuation)**					
Il1b	interleukin 1 beta	1398256_at	0.68	0.68	0.78
**Increase (Cancel)**					
Bmpr2	bone morphogenetic protein receptor, type II (serine/threonine kinase)	1376843_at	1.25	1.03	1.10
Cdkn1c	cyclin-dependent kinase inhibitor 1C	1372299_at	1.27	1.00	1.19
Corin	corin, serine peptidase	1382954_at	1.53	0.97	1.02
Cryab	crystallin, alpha B	1370026_at	1.35	1.02	1.14
Hba1 /// Hba2	hemoglobin, alpha 1 /// hemoglobin, alpha 2	1388608_x_at	1.27	1.20	1.03
Hmox1	heme oxygenase 1	1370080_at	1.34	0.85	1.09
Myh11	myosin, heavy chain 11, smooth muscle	1370896_a_at	1.29	1.45	1.02
**Decrease (Cancel)**					
Apob	apolipoprotein B	1388190_at	0.63	1.13	0.90
Gdnf	glial cell derived neurotrophic factor	1385180_at	0.76	0.95	0.86
Gucy1b3	guanylate cyclase 1, soluble, beta 3	1374389_at	0.73	0.86	0.89

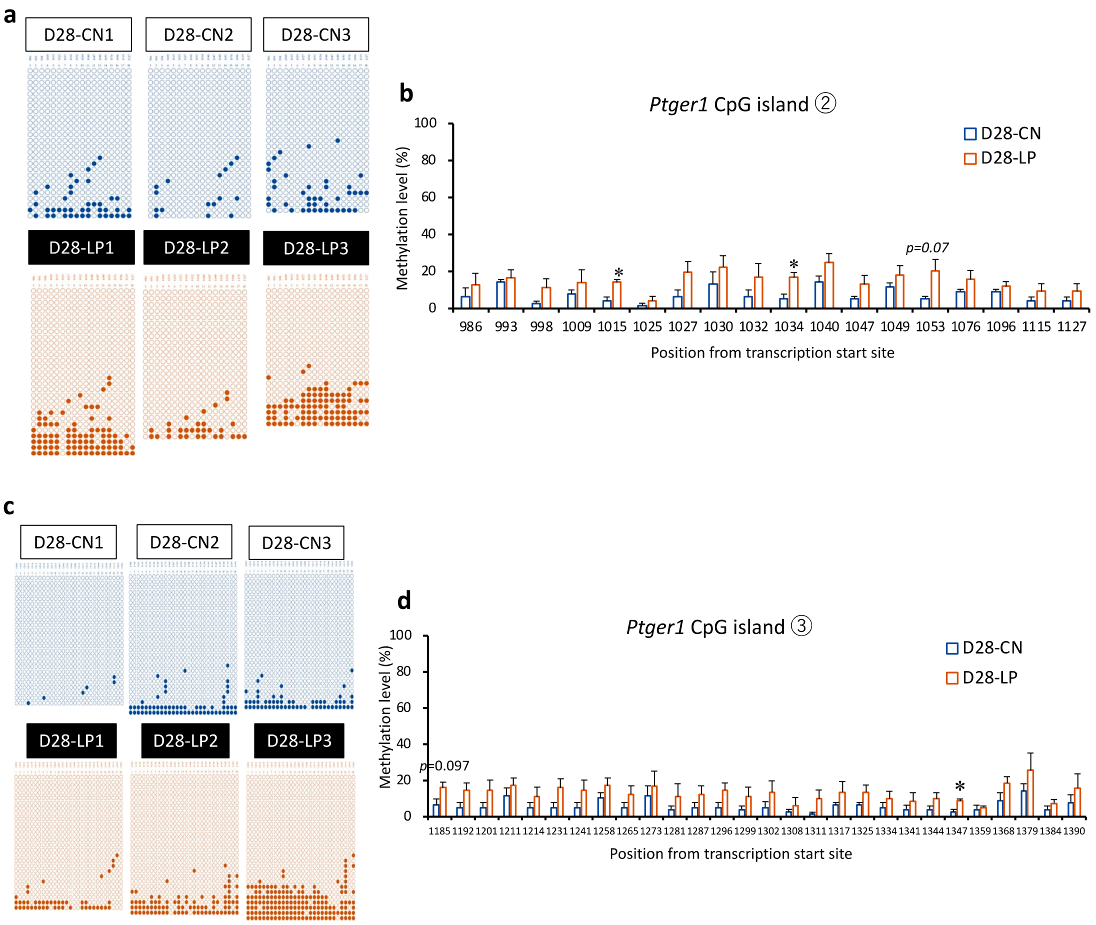

Supplemental Figure S1

Ptger1 CpG island (CpG② and CpG③) DNA methylation levels in pup (D28) kidneys of fetal exposure to maternal low protein diet. a Bisulfite sequencing analysis of the *Ptger1* CpG island ②. **b** DNA methylation levels of each CpG site in the *Ptger1* CpG island ② were calculated using the methylation data shown in (**a**). **c** Bisulfite sequencing analysis of the *Ptger1* CpG island ③. **d** DNA methylation levels of each CpG site in the *Ptger1* CpG island ③ were calculated using the methylation data shown in (**c**). The horizontal axis shows each evaluated CpG site. ○, unmethylated cytosine; ●, methylated cytosine. Data are expressed as means ± standard error (n = 3). *$p < 0.05$ vs. the D28-CN group according to a Student's t-test. *Ptger1*, Prostaglandin E Receptor 1. CpG, 5'-C-phosphate-G-3'.

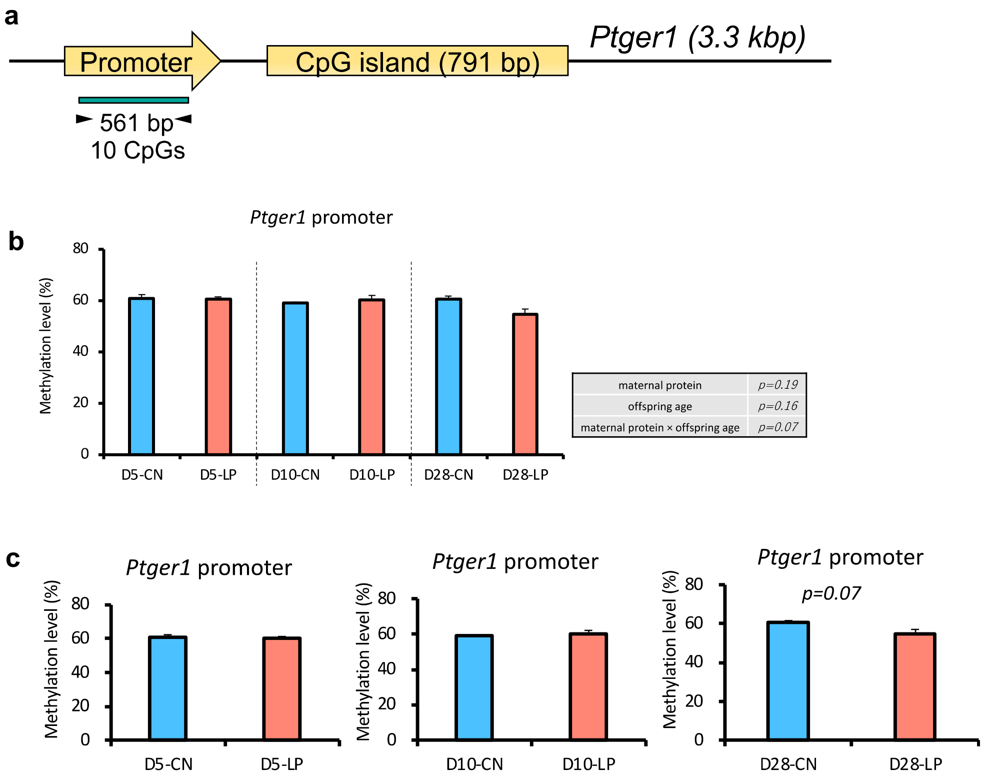

Supplemental Figure S2

***Ptger1* promoter DNA methylation levels from D5 to D28 in pup kidneys exposed to a fetal low-protein diet. a** Cartoon showing the evaluated Ptger1 promoter region. Primers were designed in the region around the TSS (-318 bp to + 243 bp). **b** DNA methylation levels of the *Ptger1* promoter over time in pup kidneys from D5 to D28 according to maternal diet and offspring age. **c** DNA methylation levels of the *Ptger1* promoter at D5, D10, and D28 according to maternal diet. Data are expressed as means ± standard error (n = 5). Statistical analysis was performed using Tukey's test (**b**) and Student's t-test (**c**).

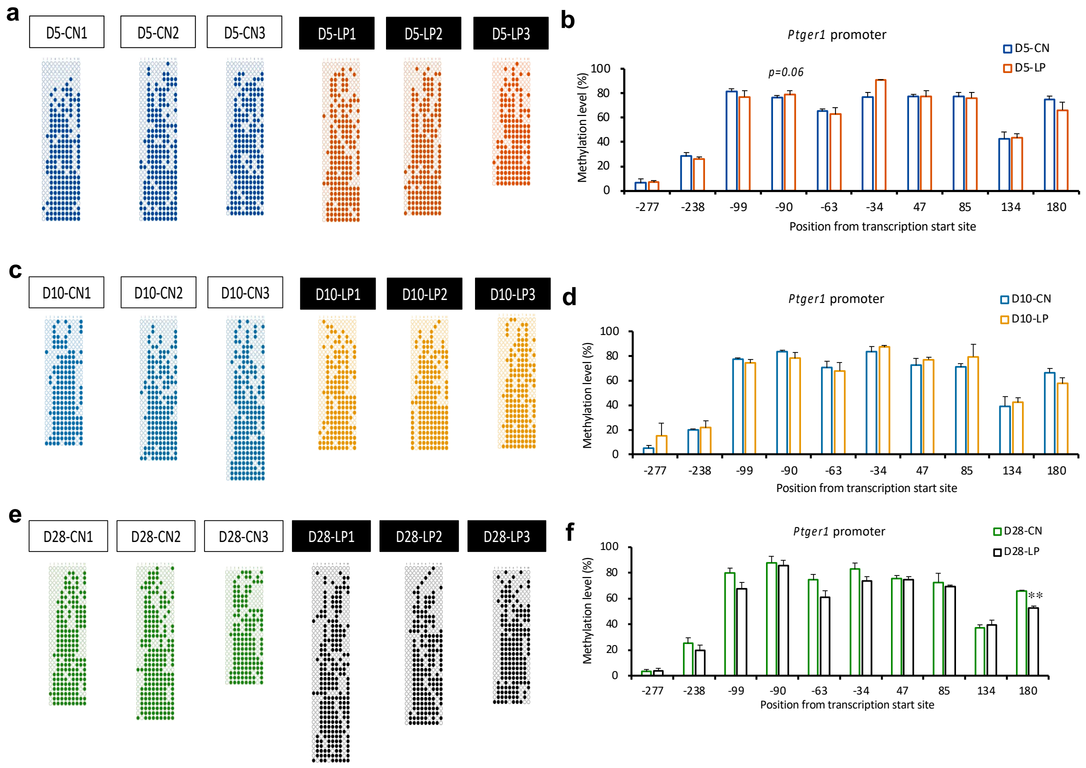

Supplemental Figure S3

***Ptger1* promoter DNA methylation levels from D5 to D28 in pup kidneys exposed to a fetal low-protein diet. a, c, and e** Bisulfite sequencing analysis of *Ptger1* promoter in D5, D10 and D28. The horizontal axis shows each evaluated CpG site. ○, Unmethylated cytosine; ●, methylated cytosine. **b, d, and f** DNA methylation levels at each CpG site in *Ptger1* promoter were calculated from the bisulfite sequencing data shown in (**a**), (**c**) and (**e**), respectively. Data are expressed as means ± standard error ($n = 5$). $^{**}p < 0.01$ vs. the D28-CN group according to a Student's *t*-test.

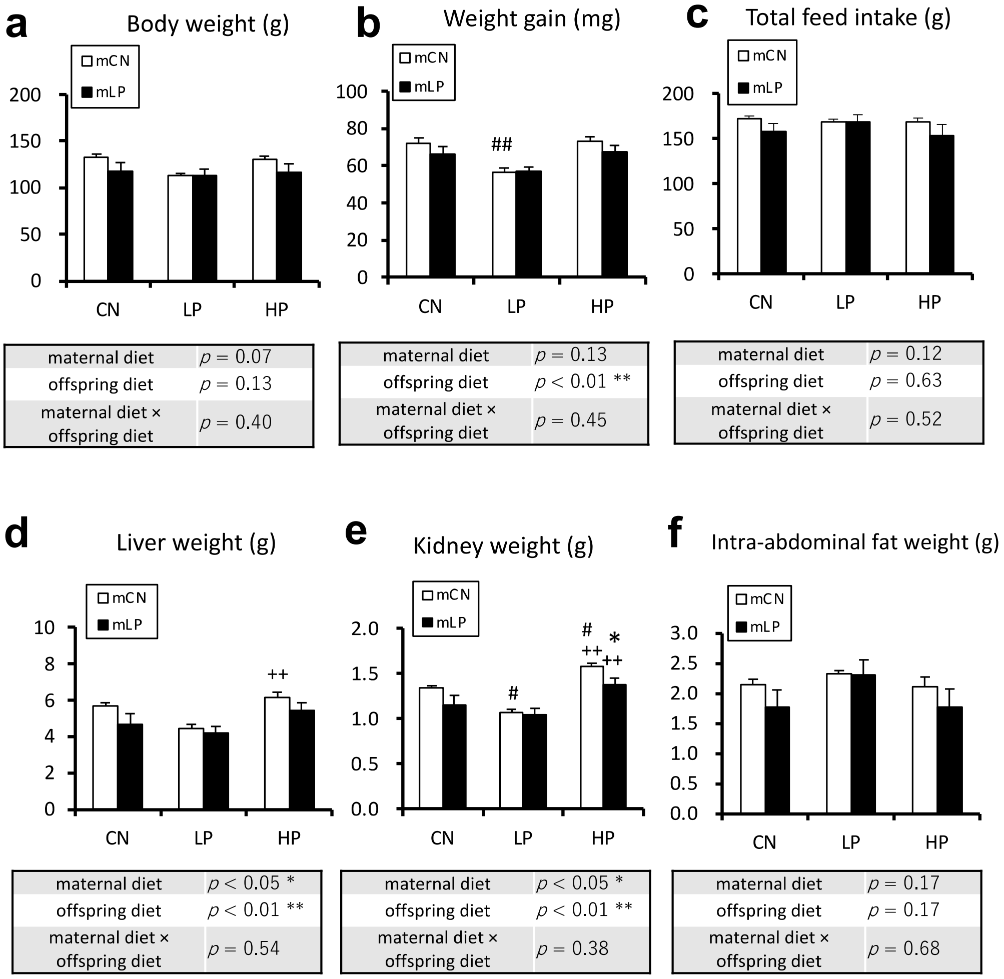

Supplemental Figure S4

Body weight, feed intake, and tissue weight either on a low-protein diet or a high-protein diet for 2 weeks after weaning (at week 4). a Body weight of adult offspring (D42) in each group. **b-c** The graphs illustrate the weight gain (**b**) or total feed intake (**c**) from the age of 4 to 6 weeks during the low-protein or high-protein diet trials. **d-f** The graphs illustrate the liver weight (**d**), kidney weight (**e**) and intra-abdominal fat weight (**f**) (total weight of mesenteric fat, retroperitoneal fat, and peritesticular fat) in 6-week-old offspring of each group. Data are expressed as means ± standard error (*n* = 5). $^{*}p < 0.05$ vs. corresponding mCN group; $^{\#\#}p < 0.01$, $^{\#}p < 0.05$ vs. corresponding CN group; $^{++}p < 0.01$ vs. corresponding LP group. Statistical analysis was performed using two-way analysis of variance followed by Tukey's test. mCN/mLP: maternal control diet/maternal low protein diet; CN/LP/HP: offspring CN/LP/HP diet.
